# Insight into antistaphylococcal effect of chlorinated 1-hydroxynaphthalene-2-carboxanilides

**DOI:** 10.5599/admet.2684

**Published:** 2025-03-26

**Authors:** Lucia Vrablova, Tomas Gonec, Petra Majerova, Andrej Kovac, Dominika Kos, Peter Kollar, Jiri Kos, Alois Cizek, Tereza Kauerova, Josef Jampilek

**Affiliations:** 1 Department of Analytical Chemistry, Faculty of Natural Sciences, Comenius University, Ilkovicova 6, 842 15 Bratislava, Slovakia; 2 Department of Chemical Drugs, Faculty of Pharmacy, Masaryk University, Palackeho tr. 1946/1, 612 00 Brno, Czech Republic; 3 Institute of Neuroimmunology, Slovak Academy of Sciences, Dubravska cesta 9, 845 10 Bratislava, Slovakia; 4 Department of Molecular Pharmacy, Faculty of Pharmacy, Masaryk University, Palackeho tr. 1946/1, 612 00 Brno, Czech Republic; 5 Department of Pharmacology and Toxicology, Faculty of Pharmacy, Masaryk University, Palackeho tr. 1946/1, 612 00 Brno, Czech Republic; 6 Department of Biochemistry, Faculty of Medicine, Masaryk University, Kamenice 5, Brno 625 00, Czech Republic; 7 Department of Infectious Diseases and Microbiology, Faculty of Veterinary Medicine, University of Veterinary Sciences Brno, Palackeho tr. 1946/1, 612 42 Brno, Czech Republic; 8 Institute of Chemistry, University of Silesia, Szkolna 9, 40-006 Katowice, Poland

**Keywords:** Lipophilicity, antistaphylococcal activity, cytotoxicity, MTT assay, chemoproteomic analysis

## Abstract

**Background and purpose:**

New compounds and innovative therapeutic approaches are trying to prevent antimicrobial resistance, which has become a global health challenge.

**Experimental approach:**

This study includes a series of twelve mono-, di- and trichlorinated 1-hydroxynaphthalene-2-carboxanilides designed as multitarget agents. All compounds were evaluated for their antistaphylococcal activity. Furthermore, MTT assay and chemoproteomic analysis of selected compounds were performed. Cytotoxicity in human cells was also tested.

**Key results:**

*N*-(3,5-Dichlorophenyl)-1-hydroxynaphthalene-2-carboxamide (**10**) demonstrated activity comparable to or higher than clinically used drugs, with minimum inhibitory concentrations (MICs) of 0.37 μM. The compound was equally effective against clinical isolates of methicillin-resistant *S. aureus*. On the other hand, compound **10** showed 96 % inhibition of *S. aureus* respiration only at a concentration of 16× MIC. Chemoproteomic analysis revealed that the effect of agent **10** on staphylococci resulted in the downregulation of four proteins. This compound expressed no *in vitro* cytotoxicity up to a concentration of 30 μM.

**Conclusion:**

From the set of tested mono-, di- and trisubstituted derivatives, it is evident that the position of chlorine atoms is decisive for significant antistaphylococcal activity. Inhibition of energy metabolism does not appear to be one of the main mechanisms of action of compound **10**; on the contrary, the antibacterial effect may likely be contributed by downregulation of proteins (especially ATP-dependent protease ATPase subunit HslU) involved in processes essential for bacterial survival and growth, such as protein, nucleotide/nucleic acid synthesis and efficient protein repair/degradation.

## Introduction

Antimicrobial resistance (AMR) is a major global health challenge of the 21^st^ century. Dangerous drug-resistant bacteria include third-generation cephalosporin-resistant *Escherichia coli* and methicillin-resistant *Staphylococcus aureus*, as well as *Klebsiella pneumoniae*, *Pseudomonas aeruginosa*, and vancomycin-resistant *Enterococcus faecalis*/*Enterococcus faecium* [1-5]. Studies published in the Lancet highlight a worrying trend in the burden of AMR among people over 70 years of age, which is critical given the rapidly ageing ‘Western’ global community. Given the high variability in the burden of AMR by location and age, it is important that interventions to prevent/reduce AMR, leading to a reduction in deaths, combine infection prevention, vaccination, minimization of inappropriate antibiotic use in agriculture and humans, and research into new antibiotics [5-7].

There are many innovative approaches to the discovery, design and development of new antimicrobial drugs [8-12]; one of them is the design of multitargeted agents, *i.e.* compounds capable of interfering with microorganisms at multiple sites and preventing them from developing resistance through multiple interventions, whether in serial or parallel action on metabolic pathways, reproduction or action on membrane systems [13-17].

Salicylamides are a typical example of such multitarget agents, which are characterized by a complex anti-invasive effect against parasites, microorganisms and cancer cells [16,18-20,21 and Refs. therein, 22-26], they even have herbicidal effects [27,28]. Hydroxynaphthalenecarboxanilides, cyclic analogues of the parent salicylanilides prepared by our team, can also be considered multitarget compounds. 1-Hydroxynaphthalene--2-carboxanilides have been investigated primarily as potential anti-infective [29-31] and anti-cancer [32,33] agents. Although it is believed that these molecules may act as proton shuttle inhibitors, *i.e.* these compounds may destroy the cellular proton gradient [34,35], salicylamides' main mechanisms of action appear to be inhibition of many enzymes [19-27, 28 and Refs. therein], including inhibition of the two-component regulatory system of bacteria [36,37].

A disadvantage of multitargeted agents is that it is difficult to precisely determine/identify their mechanisms of action. One option that could help uncover the mechanism of action of these compounds is the use of chemoproteomics, which has become a useful tool in modern drug discovery and preclinical research [38], providing critical insight into the interactions between bioactive compounds and protein targets within complex biological systems [39]. By identifying the proteins to which small molecules bind, chemoproteomics allows for mapping the mechanisms of action of potential therapeutics as well as identifying unintended off-target effects that could lead to toxicity [40]. The primary goal of chemoproteomics is to investigate how small bioactive molecules interact with proteins in their native cellular environment [41]. This is typically achieved using activity-based protein profiling (ABPP), a powerful chemoproteomics technique that uses small molecular probes to label and capture proteins in a functional state [42]. The resulting labeled proteins can then be analyzed using liquid chromatography-tandem mass spectrometry (LC-MS/MS), which provides a detailed proteomic profile of the cellular response to the compound [38,43].

Given the demonstrated improvement of anti-infective properties through chlorination [22,44-50], this study aims to elucidate the previously unreported antistaphylococcal activity of chlorinated 1-hydroxynaphthalene-2-carboxanilides. Specifically, the collection of susceptible *S. aureus* and its MRSA isolates were deliberately selected as they represent the most prevalent and clinically significant Gram-positive pathogens responsible for a spectrum of infections ranging from superficial skin lesions to severe conditions like pneumonia and sepsis [51]. To investigate the mechanism of action, not only the antistaphylococcal efficacy was evaluated, but also the impact of active derivatives on bacterial respiration (energy metabolism) and intracellular processes. The application of a contemporary chemoproteomic approach, including the comparative ABPP technique, supported the effort to determine the protein profile alterations induced in both reference *S. aureus* and MRSA strains following treatment with selected ring-substituted 1-hydroxynaphthalene-2-carboxanilides. This comprehensive study, including proteomic analysis, is expected to provide valuable insights into the molecular mechanisms underlying the antistaphylococcal effects of these novel chlorinated compounds.

## Experimental

### Synthesis

The synthetic pathway and characterization of compounds **1**-**4** were described by Gonec *et al.* [29], while compounds **5**-**13** were described in [52].

### Lipophilicity determination by HPLC

An HPLC system Agilent 1200 equipped with a DAD detector (Agilent, Santa Clara, CA, USA) was used. A chromatographic column Symmetry^®^ C_18_ 5 μm, 4.6×250 mm, part No. WAT054275 (Waters Corp., Milford, MA, USA) was used. The HPLC separation process was monitored and evaluated with EZChrom Elite software ver. 3.3.2 [53] (Agilent). Isocratic elution by a mixture of MeOH p.a. (72 %) and H_2_O-HPLC Mili-Q grade (28 %) as a mobile phase was used for the determination of capacity factor *k*. Isocratic elution by a mixture of MeOH *p.a*. (72 %) and acetate-buffered saline (pH 7.4 and pH 6.5) (28 %) as a mobile phase was used for the determination of distribution coefficients expressed as *D*_7.4_ and *D*_6.5_. The total flow of the column was 1.0 mL/min, the injection volume was 20 μL, the column temperature was 40 °C, and the sample temperature was 10 °C. The detection wavelength of 210 nm was chosen. A KI methanolic solution was used for the determination of the dead times (*t*_D_). Retention times (*t*_R_) were measured in minutes. The capacity factors *k* were calculated according to the formula *k* = (*t*_R_−*t*_D_)/*t*_D_, where *t*_R_ is the retention time of the solute, and *t*_D_ is the dead time obtained using an unretained analyte. The distribution coefficients *D*_pH_ were calculated according to the formula *D*_pH_ = (*t*_R_−*t*_D_)/*t*_D_. Each experiment was repeated three times.

### Antistaphylococcal screening

*In vitro* antistaphylococcal activity of the synthesized compounds was evaluated against three clinical isolates of methicillin-resistant *S. aureus*: clinical isolate of animal origin, MRSA 63718 (Department of Infectious Diseases and Microbiology, Faculty of Veterinary Medicine, University of Veterinary Sciences Brno, Czech Republic) [54], carrying the *mecA* gene [55]; and MRSA SA 630 and MRSA SA 3202 [54] (National Institute of Public Health, Prague, Czech Republic), both of human origin. Suspected colonies were confirmed by PCR; a 108bp fragment specific to *S. aureus* was detected [56]. All isolates were tested for the presence of the *mecA* gene encoding methicillin resistance [57]. These three clinical isolates were classified as vancomycin-susceptible (but with higher MIC of vancomycin equal to 2 μg/mL (VA2-MRSA) within the susceptible range for MRSA 63718) methicillin-resistant *S. aureus* (VS-MRSA) [54]. Vancomycin-susceptible methicillin-susceptible *S. aureus* (VS-MSSA) ATCC 29213, obtained from the American Type Culture Collection, was used as the reference and quality control strain. The minimum inhibitory concentrations (MICs) were evaluated by the microtitration broth method according to the CLSI [58,59] with some modifications. The compounds were dissolved in DMSO (Sigma, St. Louis, MO, USA) to get a concentration 10 μg/mL and diluted in a microtitration plate in a medium Cation Adjusted Mueller-Hinton (CaMH, Oxoid, Basingstoke, UK) to reach the final concentration of 256-0.0625 μg/mL. The plate was inoculated by the tested microorganisms. The final concentration of bacterial cells was 10^5^ for bacteria. Ampicillin and ciprofloxacin (Sigma) were used as reference drugs. A drug-free control and a sterility control were included. The plates were incubated for 24 h at 37 °C. After static incubation in the darkness in an aerobic atmosphere, the MIC was visually evaluated as the lowest concentration of the tested compound, which completely inhibited the growth of the microorganism. The experiments were repeated three times.

### MTT assay

Compounds were prepared as previously stated and diluted in CaMH broth for *S. aureus* to achieve the desired final concentrations. *S. aureus* bacterial suspension in sterile distilled water at 0.5 McFarland was diluted 1:3. Inocula were added to each well by multi-inoculator. Diluted mycobacteria in broth free from inhibiting compounds were used as the growth control. All compounds were prepared in duplicate. Plates were incubated at 37 °C for 24 h for *S. aureus*. After the incubation period, 10 % well volume of MTT (3-(4,5-dimethylthiazol-2-yl)-2,5-diphenyltetrazolium bromide) reagent (Sigma) was mixed into each well and incubated at 37 °C for 1 h for *S. aureus*. Then 100 μL of 17 % sodium dodecyl sulphate in 40 % dimethylformamide was added to each well. The plates were read at 570 nm. The absorbance readings from the cells grown in the presence of the tested compounds were compared with uninhibited cell growth to determine the relative percent inhibition. The percent inhibition was determined through the MTT assay. The percent viability is calculated through the comparison of a measured value and that of the uninhibited control: Viability = (OD_570E_/OD_570P_)×100, where OD_570E_ is the reading from the compound-exposed cells, while OD_570P_ is the reading from the uninhibited cells (positive control). Cytotoxic potential is determined by a percent viability of <70 % [60,61].

### Cytotoxicity assay

Cytotoxicity of the compounds was determined using an LDH assay kit (Roche, Mannheim, Germany) as described previously [31]. Human monocytic leukemia THP-1 cells (European Collection of Cell Cultures, Salisbury, UK) were exposed for 24 h at 37 °C to various compound concentrations ranging from 0.37 to 30 μM in RPMI 1640 medium. For LDH assays, cells were seeded into 96-well plates (5×10^4^ cells/well in 100 μL of culture medium) in triplicate in serum-free RPMI 1640 medium and measurements were taken 24 h after the treatment with the compounds. The maximum concentration of DMSO (Sigma) in the assays never exceeded 0.1 %. Oxaliplatin and camptothecin (Sigma) were used as reference drugs. The median lethal dose values, LC_50_, were deduced through the production of a dose-response curve. All data from three independent experiments were evaluated using GraphPad Prism 5.00 software [62].

### Proteomic study

This study utilized activity-based protein profiling (ABPP), specifically its comparative technique, to identify protein targets within the proteome of cell lysates. The experiments were conducted on the universally sensitive collection strain *Staphylococcus aureus* ATCC 29213 and MRSA SA 630 isolate [54]. Both strains were treated as described above. Prior to MS detection, proteins underwent tryptic digestion.

The bacterial pellet was homogenized in a lysis buffer containing 200 mM Tris, 150 mM NaCl, 1 mM ethylenediaminetetraacetic acid (EDTA), 1 mM sodium orthovanadate (Na_3_VO_4_), 20 mM sodium fluoride (NaF), 0.5 % Triton X-100 (pH 7.4) (Sigma), and a complete protease inhibitor cocktail (Roche). Protein precipitation was carried out overnight using 80 % ice-cold acetone (Sigma). After centrifugation, the pellets were solubilized in 8 M urea (Sigma), and protein concentrations were determined using the Bio-Rad protein assay (Bio-Rad Laboratories, Colbe, Germany). A total of 100 μg of protein was reduced with 10 mM dithiothreitol (Sigma) in 100 mM ammonium bicarbonate at 37 °C for one hour. Alkylation was performed with 15 mM iodoacetamide (Sigma) in 100 mM ammonium bicarbonate, protected from light for 30 min. Trypsin digestion (Promega, Madison, WI, USA) was performed at an enzyme-to-substrate ratio of 1:100, with overnight incubation at 37 °C.

Aliquots of purified complex peptide mixtures of 100 ng were separated using Acquity M-Class UHPLC (Waters). Samples were loaded onto the nanoEase Symmetry C18 trap column (25 mm length, 180 μm diameter, and 5 μm particle size). After 2 min of desalting/concentration by 1 % acetonitrile containing 0.1 % formic acid at a flow rate of 8 μL/min, peptides were introduced to the nanoEase HSS T3 C18 analytical column (100 mm length, 75 μm diameter, and 1.8 μm particle size). For the thorough separation, a 90 min gradient of 5 to 35 % acetonitrile with 0.1 % formic acid was applied at a flow rate of 300 nL/min. The samples were sprayed (3.1 kV capillary voltage) to the quadrupole time-of-flight mass spectrometer Synapt G2-Si (Waters) with an ion mobility option. Spectra were recorded in a data-independent manner in high-definition MSE mode. Ions with 50 to 2000 *m*/*z* were detected in both channels, with a 1 s spectral acquisition scan rate. Peak detection and processing were executed using Progenesis QI 4.0 software [63] (Waters). For peak picking, the following thresholds were applied: low-energy 320 counts and high-energy 40 counts. Precursors and fragment ions were coupled using correlations of chromatographic elution profiles in low/high-energy traces. Then, peak retention times were aligned across all chromatograms. Peak intensities were normalized to the median distribution of all ions, assuming that most signals were unaffected by the experimental conditions. Protein identification was performed in Progenesis QI 4.0 [63] utilizing the *Staphylococcus aureus* Uniprot database [64].

## Results and discussion

### Chemistry and computational ADME properties

**Scheme 1.**
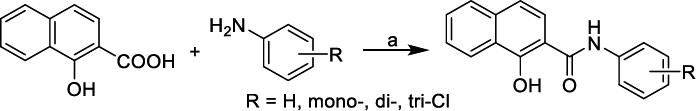
Synthesis of ring-substituted 1-hydroxynaphthalene-2-carboxanilides **1**-**13**. Reagents and conditions: (a) PCl_3_, chlorobenzene, microwave synthesis (500 W, 130 °C, 15 min).

The synthesis of the target anilides (see Scheme 1) was performed by click chemistry realized in a microwave reactor using phosphorus chloride in dry chlorobenzene from starting materials. 1-Hydroxynaphthalene-2-carboxylic acid and a suitable aniline as described in Gonec *et al.* [29] for **1**-**4** or Gonec *et al.* [52] for **5**-**13**.

All the compounds are mentioned in Table 1, where their physicochemical properties are also listed as key parameters for their drug-likeness [65]. Their lipophilicity was measured experimentally (expressed as the logarithm of the capacity factor *k* and the logarithms of the distribution coefficients *D*_pH_ at pH 6.5 and 7.4), and log *P* was predicted using the commercially available ACD/Percepta ver. 2012 program [66]. Lipinski's rule of five (Ro5) parameters [67,68] were also calculated using this software, and information about compounds related to the Veber rule was also added to clarify whether the compounds should have good oral bioavailability [68,69]. In addition to the parameters characterizing ADME, Table 1 includes the electronic *σ*_(Ar)_ parameters of the entire substituted anilide ring, characterizing the ability to withdraw or donate electrons to the molecular system. The values of *σ*_(Ar)_ are found in a wide range from 0.60 (unsubstituted derivative **1**) to 1.56 (compound **11**, R = 2,4,5-Cl).

**Table 1.** Structure of 1-hydroxynaphthalene-1-carboxanilides **1**-**13**; experimentally determined log *k*, log *D*_6.5_, log *D*_7.4_ values and predicted lipophilicity (log *P*) values, their molecular weight (MW), number of H-bond donors (HBD), number of H-bond acceptors (HBA), number of rotatable bonds (RB), topological polar surface area (TPSA), and electronic *σ*_(Ar)_ parameters of anilide ring of investigated compounds

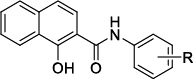											
Comp.	R	log *k*	log *D*_6.5_	log *D*_7.4_	log *P*^a^	MW^a^	HBD^a^	HBA^a^	RB^a^	TPSA^a^, nm^2^	*σ*_(Ar)_^a^
1	H	0.6084	0.5139	0.5487	4.52	263.29	2	3	2	0.49	0.60
2	2-Cl	0.6900	0.5828	0.5836	5.02	297.74	2	3	2	0.49	1.05
3	3-Cl	0.8556	0.7362	0.7227	5.25	297.74	2	3	2	0.49	0.85
4	4-Cl	0.8880	0.7640	0.7501	5.24	297.74	2	3	2	0.49	0.75
5	2,3-Cl	0.6838	0.4371	0.3870	5.76	332.18	2	3	2	0.49	1.22
6	2,4-Cl	0.7387	0.5168	0.4680	5.78	332.18	2	3	2	0.49	1.12
7	2,5-Cl	0.6717	0.3949	0.3938	5.82	332.18	2	3	2	0.49	1.22
8	2,6-Cl	0.5729	0.4516	0.4594	5.52	332.18	2	3	2	0.49	1.33
9	3,4-Cl	0.9304	0.9048	0.8909	5.99	332.18	2	3	2	0.49	1.19
10	3,5-Cl	0.9704	0.9531	0.9306	6.01	332.18	2	3	2	0.49	1.11
11	2,4,5-Cl	0.6894	0.5314	0.4864	6.31	366.62	2	3	2	0.49	1.56
12	2,4,6-Cl	0.8398	0.7254	0.6825	6.15	366.62	2	3	2	0.49	1.48
13	3,4,5-Cl	1.2387	1.0960	1.0561	6.28	366.62	2	3	2	0.49	1.46
Ro5		-	-	-	<5	<500	<5	<10	-	-	-
Veber rule		-	-	-	-	-	-	-	<10	<1.40	-

^a^ACD/Percepta ver. 2012 (Advanced Chemistry Development. Inc., Toronto, ON, Canada, 2012) [66]; Ro5 = Lipinski’s rule of five.

**Figure 1.**
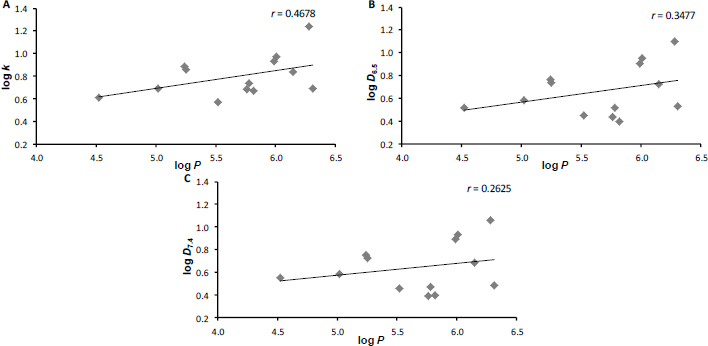
Comparison of experimentally determined values of log *k* (A), log *D*_6.5_ (B), and log *D*_7.4_ (C) with calculated log *P* (ACD/Percepta [66]) of carboxanilides **1**-**13**

Although this is a small series of compounds, it is a compact and coherent series, and interesting observations can be made due to the variability of the substitution positions in the anilide part of the molecule. The graphs in Figure 1 show the correlation between the experimental and calculated lipophilicity values. As can be seen from the values of the correlation coefficients *r*, which are in the interval 0.26-0.46 (*n* = 13), these are very poor agreements.

**Figure 2.**
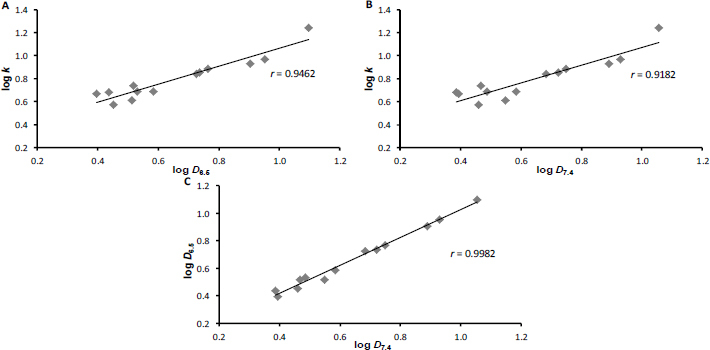
Cross-correlations of experimentally determined values of log *k* versus log *D*_6.5_ (A), log *k* versus log *D*_7.4_ (B) and log *D*_6.5_ versus log *D*_7.4_ (C) of carboxanilides **1**-**13**

The highest correlation was achieved for the comparison of log *k* versus log *P*, *i.e.* for measurements in water. At physiological pH 7.4 (*i.e.* slightly basic), the correlation is extremely low, indicating the probable role of the free phenol group in position 1 of the naphthalene scaffold and strong inter- and intramolecular interactions in the aqueous environment, which are imperceptible using the software but are essential for the behavior of this type of compounds in a biological system. Comparison of experimental log *k* versus log *D* values (Figure 2) yielded good correlations (*r* ranging from 0.91 to 0.99, *n* = 13).

**Figure 3.**
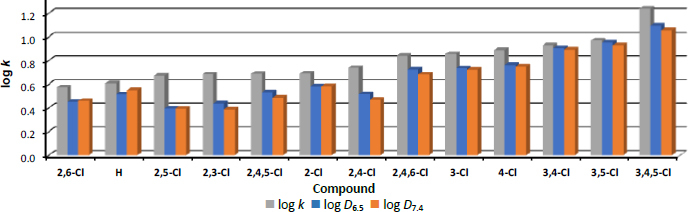
Order of individual derivatives arranged according to increasing log *k* values

Figure 3 shows the order of compounds according to increasing log *k* values. The height of the bars indicates that log *k* values are the highest compared to log *D* values, with the lowest lipophilicity values being achieved when measured at pH 7.4.

However, measurements in all three types of media confirm that, surprisingly, the unsubstituted derivative **1** is not the least lipophilic compound of the discussed series, but *N*-(2,6-dichlorophenyl)-1-hydroxynaphthalene-2-carboxamide (**8**). The most lipophilic is *N*-(3,4,5-trichlorophenyl)-1-hydroxynaphthalene-2--carboxamide (**13**), with a large jump from all other derivatives. It is clear from the graph that substitution in *ortho* positions significantly reduces lipophilicity, while substitution mainly in *meta* positions increases it.

Regarding the calculated ADME parameters listed in Table 1, Lipinski's recommendation of molecular weight <500 is met by all compounds. On the contrary, their lipophilicity, which is higher than the recommended log *P* <5 for all molecules, is contradictory. All other parameters from Ro5 and the Veber rule are met by the discussed molecules. Therefore, it is possible to assume that the compounds should not have serious absorption problems after oral administration. Much more informative results could be obtained using *in vitro* or *in vivo* experiments, which are more time-consuming and expensive [70].

### In vitro antistaphylococcal activities

All the investigated compounds were screened for their effect against *S. aureus*, as an important cause of various bacterial infections [51]. *S. aureus* ATCC 29213 was used as the reference and quality control strain, and in addition, clinical isolates of methicillin-resistant *S. aureus* (MRSA) 63718, SA 630, and SA 3202 [54] were tested. These three clinical MRSA isolates of human and veterinary origin carry the *mecA* gene, which causes their resistance [54-57]. Activities are expressed as the minimum inhibitory concentrations (MICs), see Table 2.

**Table 2.** Antistaphylococcal activity (MIC) of carboxanilides **1**-**13** compared to ampicillin (APC) and ciprofloxacin (CPX), and cytotoxicity (LC_50_) in the human monocytic leukemia cell line (THP-1) compared to oxaliplatin (OXP) and camptothecin (CMT)

Comp.	R	MIC, μM				LC_50_, μM THP-1
		*S. aureus*	MRSA 63718	MRSA SA 630	MRSA SA 3202	
1	H	>972	>972	243	121	>20 [29]
2	2-Cl	>859	>859	>859	>859	-
3	3-Cl	>859	>859	429	107	>20 [29]
4	4-Cl	>859	>859	429	107	>20 [29]
5	2,3-Cl	770	770	770	770	-
6	2,4-Cl	770	770	770	770	-
7	2,5-Cl	770	770	770	770	-
8	2,6-Cl	48.2	48.2	48.2	48.2	>30
9	3,4-Cl	770	770	96.3	385	-
10	3,5-Cl	0.37	0.37	0.37	0.37	>30
11	2,4,5-Cl	698	698	698	698	-
12	2,4,6-Cl	698	698	698	698	-
13	3,4,5-Cl	698	698	698	698	-
APC	-	5.72	45.8	45.8	45.8	0.8±0.1
CPX	-	0.75	24.2	24.2	24.2	0.7±0.09
OXP	-	-	-	-	-	1.7±0.6
CMP	-	-	-	-	-	0.16±0.07

Only *N*-(3,5-dichlorophenyl)-1-hydroxynaphthalene-2-carboxamide (**10**) showed significant antistaphylococcal activity against the susceptible strain and resistant isolates. Partial activity was also observed for carboxamide **8**. The other tested compounds were completely inactive. Therefore, no structure-activity relationships can be discussed from this set; it can only be stated that the introduction of the second chlorine substituent improves the biological activity compared to the unsubstituted derivative **1** and the monosubstituted compounds **2**-**4**. On the contrary, the third chlorine atom (derivatives **11**-**13**) again leads to a loss of activity.

Given that both active compounds have diametrically different lipophilicities (log *k* 0.9704 *vs.* 0.5729) and also distant electron-withdrawing values (*σ*_(Ar**)**_ 1.11 *vs.* 1.33), it can be summarized that only the position of both chlorine substituents significantly affects biological activity, as discussed, *e.g.* in [45,47,71-73].

However, it should be noted that the antistaphylococcal activities of both compounds against all the tested strains are similar, so a specific activity against *Staphylococcus* spp. can be speculated. A similar type of compounds - 3-hydroxynaphthalene-2-carboxanilides - was evaluated for their combined effect with ciprofloxacin and oxacillin and synergism was found in combinations with ciprofloxacin and indifference in combinations with oxacillin. From these facts, it can be concluded that the compounds have their specific mechanism of action against *S. aureus*/MRSA without any interference with the *mecA* gene and without any interaction with the cell wall. On the other hand, due to the synergism with ciprofloxacin, a possible inhibition of efflux pumps could be hypothesized [74].

### MTT assay

To contribute to the discovery of one of the possible mechanisms of action, a standard MTT assay was performed with active compound **10** in comparison with the drugs ampicillin and ciprofloxacin used as standards. The lowest multiples of MIC values that achieved inhibition of *S. aureus* ATCC 29213 viability greater than 70 % are shown in Table 3. The MTT assay can be used to assess cell growth by measuring respiration. The MTT-measured viability of bacterial cells is less than 70 % after exposure to the MIC values for each tested compound, which is considered a positive result of this assay. This low level of cell viability indicates inhibition of cell growth by inhibition of respiration [31,60,61]. Although the tested compound showed 96 % inhibition of respiration, this effect occurred only at 16× MIC, *i.e.* at a high concentration compared to the inhibitory effect on growth. Therefore, it can be assumed that the main mechanism of action is not inhibition of the respiratory chain.

**Table 3.** Lowest MIC values with at least 70 % inhibition of *S. aureus* ATCC 29213 viability (respiratory activity)

Compounds	Concentration	*S. aureus* viability inhibition, %
10	16× MIC	96.5
APC	8× MIC	90.0
CPX	32× MIC	92.8

### Cytotoxicity

Preliminary *in vitro* cytotoxicity screening of selected compounds **8** and **10** was performed using the human monocytic leukemia cell line (THP-1). Cytotoxicity was expressed as LC_50_ value (lethal concentration for 50 % of the cell population), see Table 2. Treatment with 30 μM did not lead to a significant lethal effect on THP-1 cells (*e.g.* the LC_50_ values of oxaliplatin and camptothecin were 1.7±0.6 and 0.16±0.07 μM, respectively). For comparison, the LC_50_ values of ampicillin and ciprofloxacin were 0.8±0.1 and 0.7±0.1 μM, respectively. Based on these observations, it can be concluded that both tested agents can be considered non-toxic compounds.

Previously, compounds **1**, **3** and **4** were also tested for their potential cytotoxic activity in THP-1 cells. None of them induced significant lethal effects by any tested concentrations up to 20 μM [29]. Thus, compounds **1**, **3** and **4** could also be considered non-toxic substances.

### Chemoproteomic analysis

In order to gain a deeper understanding of what happens in the staphylococcal cell after exposure to these chlorinated derivatives, a primary chemoproteomic study was performed using the ABPP approach with LC-MS/MS detection. The discussed dichlorinated derivatives were tested against *S. aureus* ATCC 29213 strain and MRSA SA 630 isolate: compound **10** with high antibacterial activity and compound **9**, a molecule with no antibacterial effect for comparison, as these xenobiotics cause cell stress but do not lead to bacterial cell death. In addition, the experiment was performed by culturing *S. aureus*/MRSA without the addition of the tested molecules (control sample, CN). In this way, the following information was obtained: (i) about the representation of proteins in native bacteria (CN); (ii) the change in the protein profile after exposure to an inactive substance (CN *vs.* **9**); and (iii) changes in the protein profile induced by the active agent (CN *vs.* **9** *vs.* **10**). This allows us to filter out misleading information that does not lead to a substantial effect on the viability of the studied staphylococci.

The results of the proteomic analysis are interpreted graphically as heatmaps and volcano plots, where differences in protein levels between samples are clearly visible. Both types of plots are created using the web platform MetaboAnalyst 6.0 [75], and brief information about the function of proteins and their assignment to specific biochemical pathways was obtained from the freely available Uniprot database [64]. From each protein set of approximately 100 proteins, the 50 proteins exhibiting the most significant changes were selected for the heatmaps. Each colored cell on the map corresponds to the value of the protein peak area in the data table, with samples in rows and proteins in columns.

**Figure 4.**
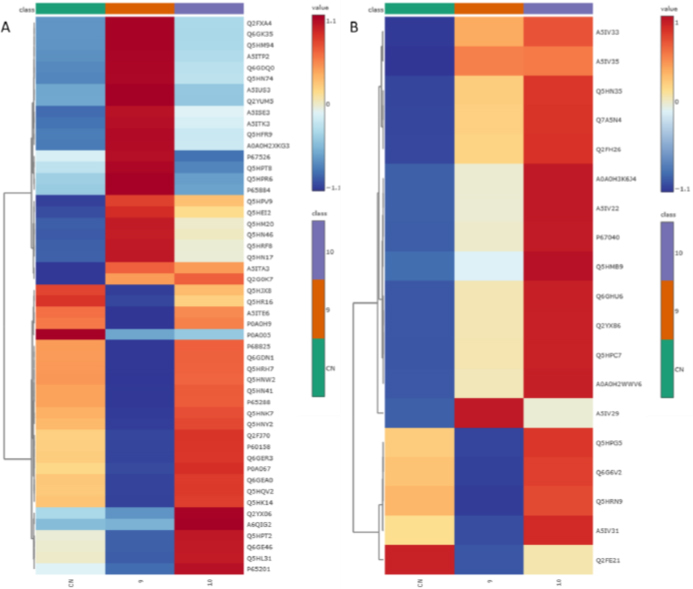
*S. aureus* heatmap comparing proteomic profiles in control sample (CN), sample with antibacterially inactive compound **9** and sample with antibacterially active agent **10** (**A**), and MRSA heatmap comparing protein expression in control sample (CN), sample with inactive compound **9** and sample with active agent **10** (**B**)

The heatmap in Figure 4A shows the differences in protein expression between the control *S. aureus* sample (*i.e.* without treatment with any compound, CN) and *S. aureus* treated with antistaphylococcal inactive compound **9** and active agent **10**. It is clear from the heatmap (Figure 4A) that inactive **9** caused changes in the proteome more significantly compared to active **10**. The active agent specifically caused downregulation of the following proteins (their function is listed in Table 4): Probable tautomerase SAV1363 (P67526), ribonuclease J2 (Q5HPR6), ATP-dependent protease ATPase subunit HslU (Q5HPT8), adenylosuccinate synthetase (P65884). Arsenate reductase (P0A005) was reduced by both derivatives to a similar level. The heatmap in Figure 4B comparing the MRSA control sample with samples **9** and **10** shows smaller protein sets compared to Figure 4A. Compared to the control sample, agent **10** caused downregulation of only one protein – Uncharacterized oxidoreductase SAUSA300_2422 (Q2FE2), which was also more significantly downregulated by inactive compound **9**. In addition, compound **9** caused a decrease in the expression of four other proteins (bottom of the heatmap): Large ribosomal subunit protein uL2 (A5IV31), Uncharacterized lipoprotein SAS2259 (Q6G6V2), Dihydroneopterin aldolase (Q5HRN9), and aminoacyltransferase FemB (Q5HPG5).

**Table 4.** Downregulated proteins detected after treatment with active agent **10** and their functions in bacteria.

Protein	Function
Probable tautomerase SAV1363 (P67526)	Protein involved in processes mediating tautomerization, microbial metabolism in various environments, and degradation/detoxification of aromatic compounds (xenobiotics) [64,76].
Ribonuclease J2 (Q5HPR6)	Metalloprotein (cofactor two Zn^2+^ ions) exhibiting 5'-3' exonuclease and endonuclease activity, respectively, which plays a role in rRNA maturation. It is also involved in mRNA synthesis and/or degradation. It affects bacterial viability through the regulation of gene expression at the RNA level [64].
ATP-dependent protease ATPase subunit HslU (Q5HPT8)	ATPase subunit of the proteasome-like degradation complex with chaperone activity. It is involved in the vital function of ridding the cell of damaged, misfolded or abnormal proteins (*e.g.* due to stress induced by antibiotic treatment) as well as controlling the levels of regulatory proteins [64,77].
Adenylosuccinate synthetase (P65884)	Key role in the de novo biosynthetic pathway of purine nucleotides essential for bacteria (basic cellular processes, DNA/RNA synthesis and energy metabolism). It catalyzes the first key step in the synthesis of adenosine monophosphate (AMP) from inosine monophosphate (IMP). It requires the presence of Mg^2+^ ions for its function [64].

**Figure 5.**
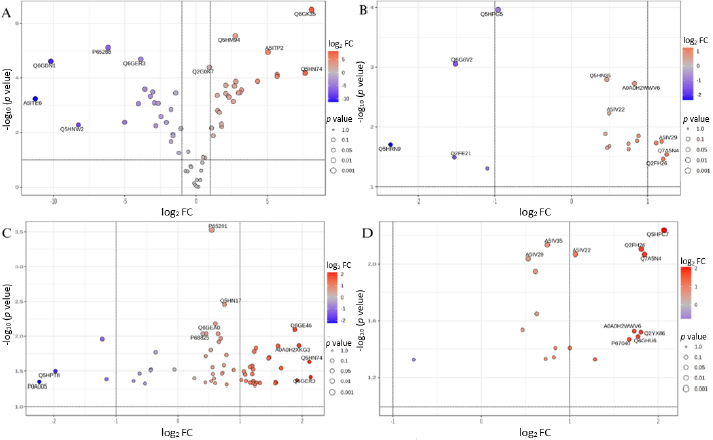
Volcano plot of fold changes (FC) in protein expression in *S. aureus* between control sample and compound **9** (A), between control and agent **10** (C), changes in expression in MRSA between control and compound **9** (B), between control and agent **10** (D)

The volcano plot of expression changes between samples shows several of the most significant differences between the control and samples **9** and **10**. Inactive compound **9** caused downregulation of significantly more proteins (Figure 5A) compared to active agent **10** (Figure 5C). When comparing the scales on the x-axis, it is clear that the proteins in Figure 5A show several times greater changes in expression or inhibition, respectively, than those in Figure 5C. Most of the proteins that were affected by compound **9** are located in the region of the graph, showing high statistical significance, while the proteins affected by the effect of compound **10** are seen in the bottom parts of the graph. It is important to note that when compared to the heatmap (Figure 4A) showing the same compounds, four significant proteins were identified. The volcano plot shows that of these proteins, only the ATP-dependent protease ATPase subunit HslU (Q5HPT8) shows the most significant change. The others were downregulated but not significantly compared to the native state of the proteins in the control sample of *S. aureus*. The MRSA samples shown in Figure 5B (control *vs.* **9**) and Figure 5D (control *vs.* **10**) show a smaller difference in the extent of changes compared to the volcano plots in Figures 5A and 5C. At the same time, the MRSA proteome was significantly more affected by compound **9** than by agent **10**. From the heatmap (Figure 4B) correlated with the given compounds, only the protein uncharacterized oxidoreductase SAUSA300_2422 (Q2FE2) was identified as downregulated, but its change was assessed as insufficiently significant (Figure 5D).

The proteins that were found to be downregulated after exposure to active agent **10** and their significance/function for bacterial viability are listed in Table 4.

If certain proteins are downregulated in bacteria even after treatment with an antibacterially inactive compound (high MIC value), as observed with compound **9** (compare 770 μM *vs.* 0.37 μM for agent **10**), this suggests that decreased expression of these proteins does not significantly impact bacterial growth. While treatment with antibacterially inactive compounds induces stress [78-80], it also triggers the expression (upregulation) of alternative proteins that help maintain bacterial viability. Consequently, inactive compound **9** had a much greater impact on the bacterial proteome than active agent **10**. In contrast, the active agent **10** induces fewer proteomic changes (downregulation) but selectively eliminates proteins essential for bacterial survival.

## Conclusions

Twelve chlorinated 1-hydroxynaphthalene-2-carboxanilides were synthesized by microwave synthesis and tested for their antistaphylococcal activity against reference *S. aureus* ATCC 29213 and three clinical MRSA isolates carrying the *mecA* gene. Only one compound, *N*-(3,5-dichlorophenyl)-1-hydroxynaphthalene-2-carboxamide (**10**), was significantly active against all *Staphylococcus* sp. The MICs were the same against all strains (0.37 μM). From the set of tested mono-, di- and trisubstituted derivatives, it is evident that the position of the chlorine atoms is crucial for antistaphylococcal activity. This compound showed no lethal activity (LC_50_) against the human monocytic leukemia cell line (THP-1) up to a concentration of 30 μM. Compound **10** showed 96 % inhibition of *S. aureus* respiration at a concentration of 16× MIC. Thus, it has a significant effect, but at high concentrations, suggesting that interference with energy metabolism is not the primary mechanism of action. Proteomic analysis performed with antistaphylococcal effective agent **10** and inactive isomer **9** revealed that inactive derivative **9** alters the proteome of staphylococci more significantly than active compound **10**. The effect of agent **10** on *S. aureus* resulted in the downregulation of four proteins: probable tautomerase SAV1363, ribonuclease J2, adenylosuccinate synthetase, and especially ATP-dependent protease ATPase subunit HslU, involved in essential processes for bacterial survival and growth, such as syntheses of proteins, nucleotides/nucleic acids, and efficient protein repair/degradation. It can be assumed that this downregulation probably contributes to the antibacterial effect. The effect of compound **10** on the proteome in a clinical MRSA isolate remains unclear despite several different experiments/measurements and will require further investigation.
